# Molecular World Today and Tomorrow: Recent Trends in Biological Sciences

**DOI:** 10.3390/ijms25053068

**Published:** 2024-03-06

**Authors:** Wajid Zaman

**Affiliations:** Department of Life Sciences, Yeungnam University, Gyeongsan 38541, Republic of Korea; wajidzaman@yu.ac.kr

## Introduction

Various molecular techniques based on omics (transcriptomics, proteomics, genomics) and phylogenetics have been applied in the field of biological sciences. Molecular dynamics and approaches have evolved into various quantitative tools that allow researchers from multiple disciplines to design different studies. The molecular-based techniques can be comprehensive and systematic, as they allow for the identification and resolution of genetic differences, molecular docking, and the construction of prediction models of ecological niches and taxonomic ranks [1,2]. The investigation of genomics, proteomics, and phylogenetic techniques requires the utilization of a novel class of DNA elements such as microsatellites from mitochondria and chloroplast and retrotransposons, resulting in genetic variations using molecular data [3]. In addition, the advantages and limitations of molecular approaches have been well studied and acknowledged. The combination of molecular phylogenetic and omics techniques as well as expression and pathway analyses may greatly increase our capacity to understand and develop new molecular mechanisms and stress responses in biological systems [4,5]. Furthermore, these techniques offer extensive opportunities for researchers to develop targeted therapy approaches and disease diagnoses using molecular data. It is necessary to evaluate and explore how data from diverse molecular techniques can be applied to different biological studies. The study and applications of molecular approaches hold significant potential for advancing genomics, proteomics, and phylogenetic techniques in biological sciences.

The molecular world is a fascinating field where scientists are constantly discovering new complex systems guiding life in the dynamic field of biological sciences. The topic of this Special Issue, “Molecular World Today and Tomorrow: Recent Trends in Biological Sciences,” explores the most recent advancements in this rapidly evolving subject while highlighting knowledge gaps and outlining a course for future research. The last ten years have seen incredible progress in our understanding of the molecular details of living things. Researchers have uncovered hitherto undiscovered information regarding cellular functions, signaling pathways, and the molecular causes of diseases thanks to developments in imaging technologies, genomics, and proteomics [6,7,8]. Our understanding of complicated biological phenomena has advanced even further with the combination of artificial intelligence and big data analytics [9,10]. Nevertheless, there remains a complex reality hidden behind these victories, and there are still many unanswered questions. New questions arise when we peel back the layers of intricacy, calling scientists to delve deeper and to continue learning.

Our Special Issue “Molecular World Today and Tomorrow” provides proof of the vitality and dynamism of the biological sciences today. The contributors shed light on the routes leading to the core of molecular biology through an extensive collection of research and review papers. As we read these pages, we are reminded that the molecular world is not only a field of study, but also a source of inspiration for the discoveries that will mold the sciences related to living things in the future.
